# Clinical and genetic analysis of two wolfram syndrome families with high occurrence of wolfram syndrome and diabetes type II: a case report

**DOI:** 10.1186/s12881-020-0950-4

**Published:** 2020-01-14

**Authors:** Maryam Sobhani, Mohammad Amin Tabatabaiefar, Soudeh Ghafouri-Fard, Asadollah Rajab, Asal Hojjat, Abdol-Mohammad Kajbafzadeh, Mohammad Reza Noori-Daloii

**Affiliations:** 1 Blood Transfusion Research Center, High Institute for Research and Education in Transfusion Medicine, Tehran, Iran; 2 Department of Genetics and Molecular Biology, School of Medicine, Isfahan University of Medical Sciences, Isfahan, Iran; 3 Pediatric Inherited Diseases Research Center, Research Institute for Primordial Prevention of Non-Communicable Disease, Isfahan University of Medical Sciences, Isfahan, Iran; 4 Department of Medical Genetics, Shahid Beheshti University of Medical Sciences, Tehran, Iran; 5 Iranian Diabetes Society, Tehran, Iran; 6 Pediatric Urology Research Center, Department of Pediatric Urology, Children’s Hospital Medical Center, Tehran University of Medical Sciences, Tehran, Iran; 7 Department of Medical Genetics, School of Medicine, Tehran University of Medical Sciences, Tehran, Iran

**Keywords:** Wolfram syndrome, WFS1, Gene, Iran, Diabetes

## Abstract

**Background:**

Mutations of the *WFS1* gene are responsible for most cases of Wolfram syndrome (WS), a rare, recessively inherited neurodegenerative disorder characterized by juvenile-onset non-autoimmune diabetes mellitus and optic atrophy. Variants of *WFS1* are also associated with non-syndromic hearing loss and type-2 diabetes mellitus (T2DM). Our study adds to literature significant associations between WS and T2DM.

**Case presentation:**

In this study, we analyzed the clinical and genetic data of two families with high prevalence of WS and T2DM. Genetic linkage analysis and DNA sequencing were exploited to identify pathogenic variants. One novel pathogenic variant (c.2243-2244insC) and one known pathogenic (c.1232_1233delCT) (frameshift) variant were identified in exon eight of *WFS1* gene.

**Conclusions:**

The mutational and phenotypic spectrum of WS is broadened by our report of novel *WFS1* mutation. Our results reveal the value of molecular analysis of *WFS1* in the improvement of clinical diagnostics for WS. This study also confirms the role of *WFS1* in T2DM.

## Background

Since its first report in 1938, Wolfram syndrome (WS) and its genetic analysis still remain challenging and valuable. Wolfram and Wagener first introduced four WS cases with a description of concomitant juvenile-onset diabetes mellitus (DM) and optic atrophy (OA) [1]. WS as an uncommon autosomal recessive neurodegenerative disorder, described by additional main signs and symptoms including: diabetes insipidus, deafness and urinary tract abnormalities [2–4]. Although there are currently no effective treatments that can delay or reverse the progression of WS, the use of careful clinical monitoring and supportive care can help relieve the suffering of patients and improve their quality of life [5]. This syndrome has been firstly associated with mutations in the *WFS1* locus on 4p16 [6]. More recently, a second locus (4q24) containing a gene designated as *WFS2* (*CISD2*) was found to be associated with a small number of affected Jordanian families [7].

The *WFS1* gene mutations account for most cases of WS. The encoded protein has a protecting function against endoplasmic reticulum (ER) stress. WFS1 protein is a trans-membrane protein known as an unfolded protein response which diminishes ER stress response in cells [8, 9]. Fonseca et al. described a vital function of WFS1 in protecting secretary cells from death by its negative feedback regulatory loop of the ER stress signaling system [10]. ER has a key role in the biosynthesis of secretory proteins, redox regulation and many other pivotal cell processes [11, 12]. It has been suggested that stress-related deterioration of β cell function caused by ER, starts at the beginning of WS patient’s life [13]. Previous mutation analyses have been unable to identify clear genotype-phenotype correlations in WS cases, since genetic heterogeneity and small numbers of patients have made analyses difficult. Several reports suggest that clinical expression of WS is complete in patients harboring inactivating mutations only [14–16] and clinical features are likely to occur at a relatively earlier age [15]. Patients carrying one or more missense variants may present with a relatively milder phenotype [17–19].

Mutations in the *WFS1* gene have been more frequently detected in exon 8, also in exons 3, 4, 5 and 6 [20]. Since common variants of *WFS1* correlate with type 2 diabetes (T2DM) risk, studying this rare disease may also have relevance to the pathogenesis of T2DM [21].

In the present study, we introduce a novel mutation in the *WFS1* gene which causes WS. In addition, we report five Iranian familial cases of WS in an extended family with significant prevalence of diabetes from Yazd, a central region of Iran.

## Case presentation

**Fig. 1:**
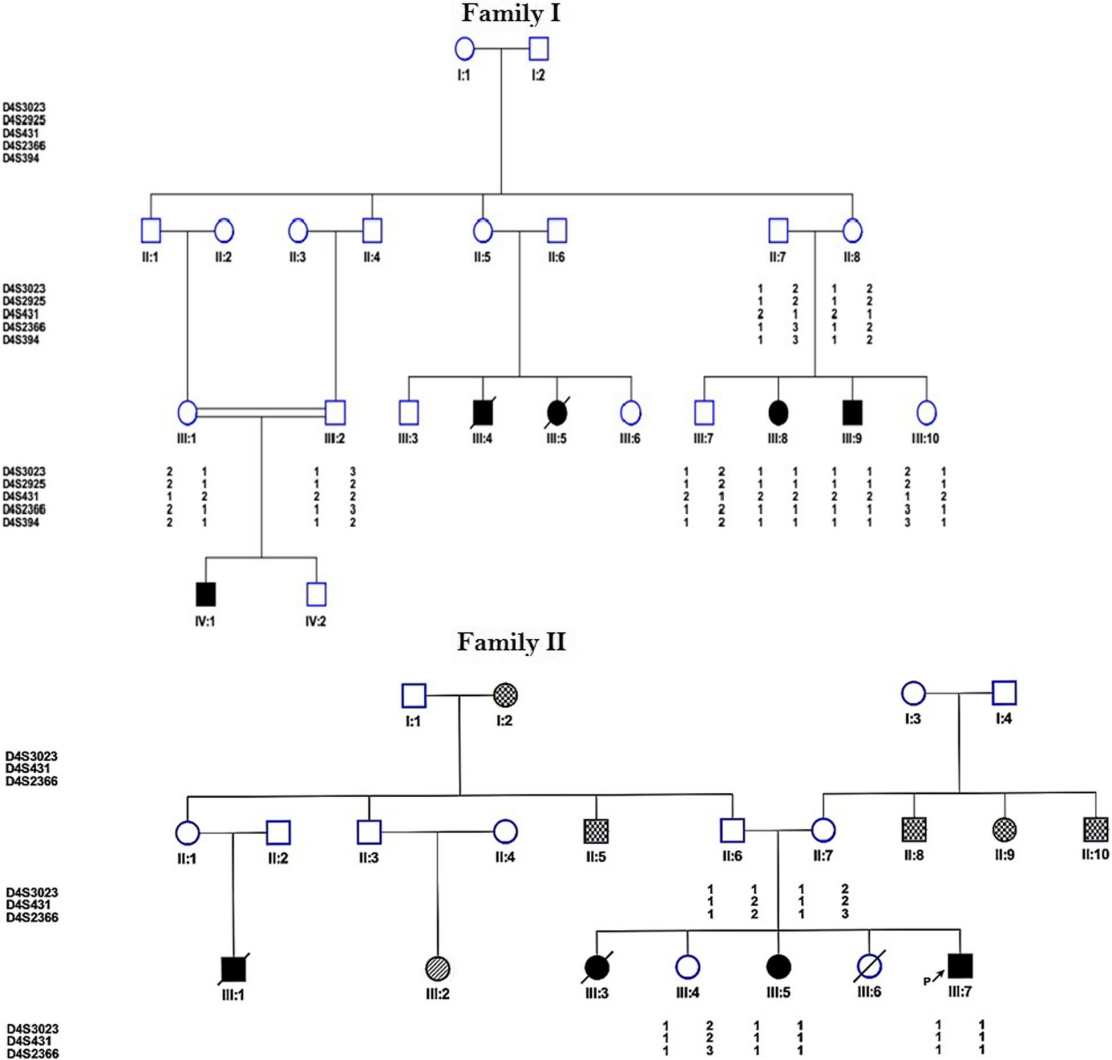
Pedigree of WS families showing the results of linkage analysis and haplotyping of Wolfram syndrome locus markers at 4p16.1. Filled squares in black denote WS individuals. In family II spotted shapes designates diabetes type 2 and the stripped shape indicates mental retardation

The study group comprised two large Iranian pedigrees with nine WS cases and first-degree relatives of the patient from two different central provinces including Yazd and Kerman provinces in 2017. Family pedigrees are shown in Fig. 1. Our data concerning clinical manifestations of WS were retrospectively gathered from patient file records. They included age of T2DM onset, familial aggregation, and other cardinal manifestation of this syndrome with special emphasis on T2DM family background in the pedigree.

### Clinical examinations

All affected individuals and non-affected family members were assessed by a group of physicians specialized in urology, otorhinolaryngology, neurology, endocrinology and ophthalmology. Complete neurological and urological assessments included detailed physical examination, urine analysis, urine culture and urinary tracts ultrasonography as described previously [22].

### Sampling and DNA extraction

This study was approved by the Institutional Review Boards of Tehran University of Medical Sciences. Written consent forms were signed by all patients or their parents. DNA was isolated from peripheral blood samples of all study participants (patients and accessible family members) using QIAamp DNA Blood kit (Qiagen, Germany). Quality of extracted DNA was assessed by a spectrophotometer (nanodrop 2000c, Thermo scientific, USA) and electrophoresis on agarose gel.

### Simulation and linkage analysis

To determine statistical power of the families Slink was calculated by FastSLink v2.51 software from easyLINKAGE Plus v5.05. Five profit STR markers were selected through NCBI Map viewer (D4S2366, D4S431, D4S3021, D4S2925 and D4S394) according to physical distance and heterozygosity and primers were selected from STS probe. Touch down PCR was carried out with initial annealing temperature beyond the probable melting temperature of the primers and gradual transitions to lower temperatures [23]. Markers were amplified according to our previous study [22]. PCR products were run on an acrylamide gel and silver staining was performed. Two-point and multipoint LOD scores were calculated by SuperLink v1.6 and GeneHunter, respectively. Haplotype was drawn by haplopainter v.1.043.

### *WFS1* gene sequencing

Exons and exon-intron boundaries of all coding regions were amplified with a set of 14 pairs of primers as described before [22]. PCR products were bi-directionally sequenced on an automated sequencer 3730xl (Applied Biosystems, Foster City, USA). Sequences were aligned with NM_006005 and NG_011700.1 for detecting exonic and splice site variants.

## Results of clinical assessments

### Family I

Clinical examinations in all the studied patients showed an average age of diagnosis for T2DM to be 6.4 years old with poor insulin control. Optic atrophy was verified in all patients but patient VI-1, who was still young the age of seven. The bilateral high-frequency sensorineural hearing loss was also evident. The phenotype characteristics of the three cases are as follows:

#### Case III:8

The patient was a 24-year-old female, born to a first-cousin couple. She experienced no special complication during pregnancy and delivery. The patient is the second child with three siblings. She was a known diabetic case from the age of 6, receiving insulin with poor compliance to treatment. Her ophthalmic evaluation revealed bilateral optic atrophy, decreased vision and peripheral constriction of the visual field. Her visual acuity was reduced to 20/80 on both sides. At the age of 17, she started complaining about polyuria, nocturia, and incontinency, and since 5 years ago she has had voiding dysfunction and has used clean intermittent catheterization (CIC) three times a day. Urine analysis and the blood test confirmed the diagnosis of diabetes insipidus. Renal ultrasonography showed pelvicalyceal dilatation in both kidneys and bilateral grade IV hydronephrosis along with dilation in proximal of ureters and bladder enlargement secondary to polyuria. Urodynamics study confirmed atonic bladder. Audiometric studies showed the bilateral high-frequency sensorineural hearing loss at the age of 16. Familial aggregation of T2DM and WS is seen in the family.

#### Case III:9

**Fig. 2:**
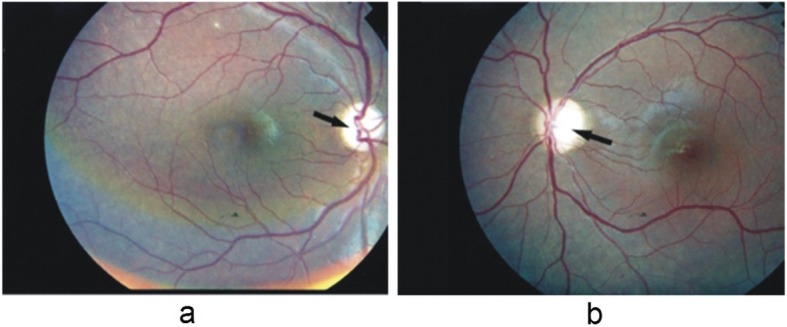
Photographic image of the right (**a**) and left (**b**) eyes of case 2 (III-9) showing optic atrophy without diabetic retinopathy

The patient was 21 years old and, was diagnosis with T2DM at 7 years of age. The same dynamics of disease progress as his affected sister had occurred. He had been experiencing decreased vision for several years. On funduscopic examination, bilateral optic atrophy without diabetic retinopathy was confirmed (Fig. 2). His visual acuity was 20/120 in both eyes. According to renal ultrasonography results, he had pelvicalyceal dilatation in both kidneys equal to grade III hydronephrosis with dilation in proximal of ureters and bladder enlargement. He and his brother (III:7), aged 23, had severe neurological problems**.**

#### Case IV:1

**Fig. 3:**
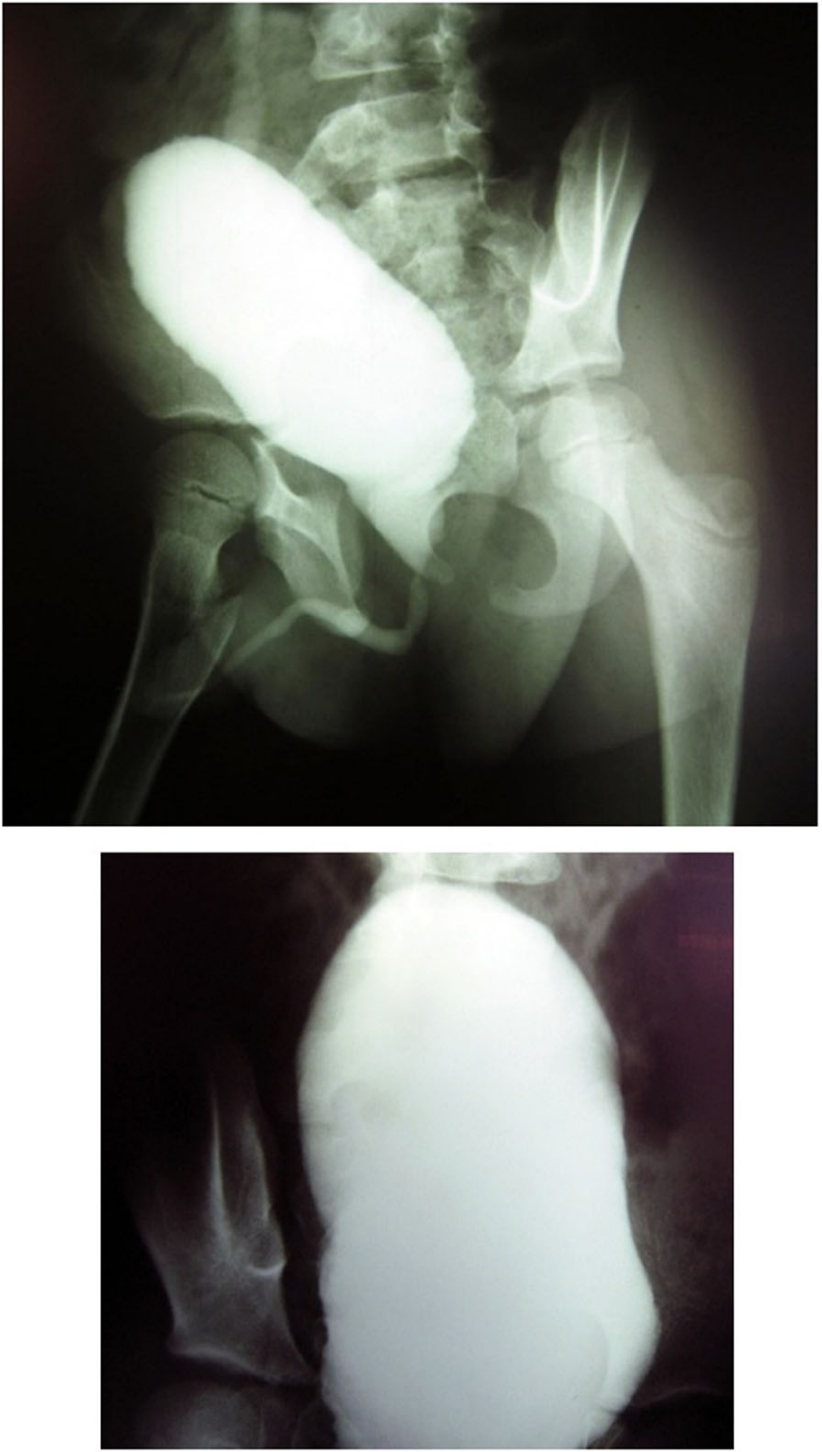
VCUG confirms right vesicourethral reflux and large bladder from case 3 (patient IV:1)

The patient was a six-year-old boy born to a first-cousin couple (Fig. 1). His postnatal period was uneventful. However, the mother noticed he was straining during micturition. Thus, he was referred to the urologist for circumcision. Urinary tract ultrasound (US) examination confirmed mild fullness of the left kidney. Follow-up annual US showed mild to moderate hydroureteronephrosis with no obstruction on renal isotope scan. He was under the care of the pediatric endocrinologist for management of T2DM since the age of 4 years and finally diagnosed with WS. Recent cystogram confirmed right vesicoureteral reflux with neuropathic bladder and high post-voiding urine residue (Fig. 3). He is under the care of a pediatric urologist. The family history was positive for T2DM and severe depression. The first child in this family was aborted after 8 weeks of pregnancy with unknown etiology (not indicated in Fig. 1).

### Family II

Four patients had been diagnosed with WS in this family. The proband (III:7) with symptoms of hyperglycemia and polyuria was diagnosed as having T2DM at the age of three. Since then progressively visual loss appeared at 14 years of age. In this patient, the visual impairment prolonged to the present time at the age of 8 and was associated with the occurrence of optic atrophy. Bilateral hydronephrosis and neurogenic bladder were detected by ultrasound evaluation.

The proband’s elder sister (III:3) had also been diagnosed as WS. The age at onset of T2DM for her was seven-year-old. Although the age of the patient at the time of atrophic optic nerve detection was 12, her gradual loss of visual acuity had been observed formerly. She underwent dialysis due to severe chronic kidney disease and consequently she died at the age of 15. The third sibling (III:5) of the present family had also been diagnosed with WS with similar disease progress. She was diagnosed with diabetes at the age of seven. She received hemodialysis over a year and finally passed away following a hypoglycemic seizure attack. Likewise, one of the cousins of this case also passed away at the age of 12-year-old due to WS. It is notable that there is a high occurrence of T2DM among the family members (I:2, II:5, II:8, II:9, II:10, and III:2). In our reported family the mean age at onset of T2DM and optic nerve atrophy were 6.4 and 7 years, respectively. High frequency bilateral sensorineural deafness was diagnosed in all cases.

## Results of molecular study

According to homozygosity for all markers studied in all patients with WS, the family was linked to the locus *WFS1*. Before genotyping of STR markers to determine the statistical power of the families, SLINK value was calculated. The maximum SLINK based on existing samples was 3.3, the maximum two points LOD score was 2.97 and the maximum multipoint LOD score for the D4S394 marker was 2.9. Family II was too small to calculate SLINK and LOD score, so just haplotyping was performed.

**Fig. 4:**
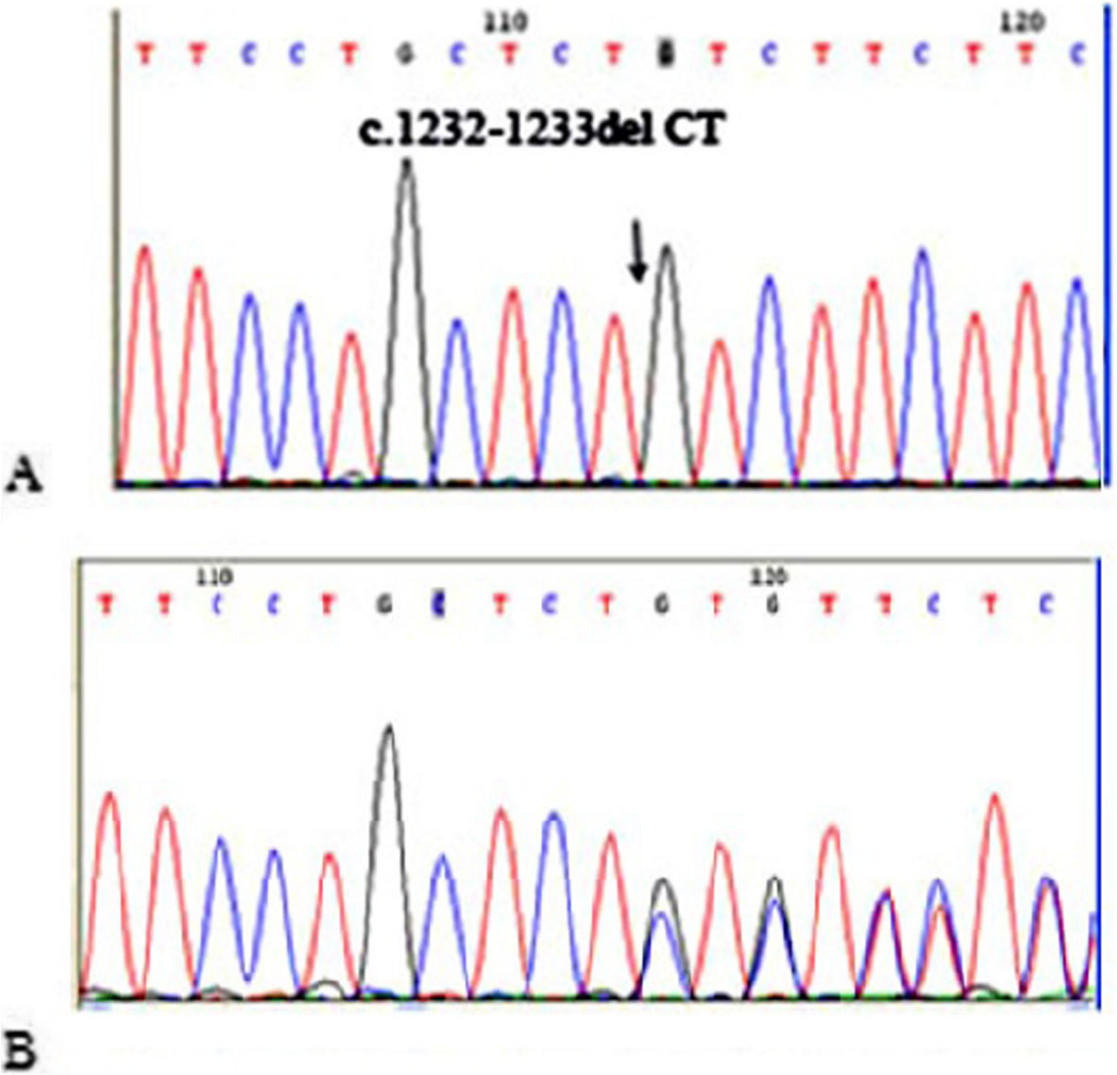
**a** WFS1 exon 8 forward sequences of a homozygote patient (IV-1), **b** one of his parents (III-1)

*WFS1* gene sequencing showed several variants (Table 1) including a single frameshift variant c.1232_1233delCT found in exon 8 (Fig. 4). Patients were homozygous for this mutation but parents and healthy siblings were heterozygous or homozygous for the wild-type allele. This two base pair deletion creates a premature stop signal at codon 132 downstream of residues 541 of the wolfram protein (S411 fs*541). Details of pathogenic analysis are provided in Additional file 1.

**Table 1:** Variants identified in *WFS1* gene sequencing

Exon 8	Exon 6	Exon 5	Exon 2	Exon 1
rs1801206	rs1801213	rs9998519	rs28420833	rs6830765
rs734312	rs71524359	rs10010131		
rs1801214	rs11725494			
rs71532864	rs11725500			
rs1046314				
rs1046316				
rs1046319				
rs1046320				
rs1046317

**Fig. 5:**
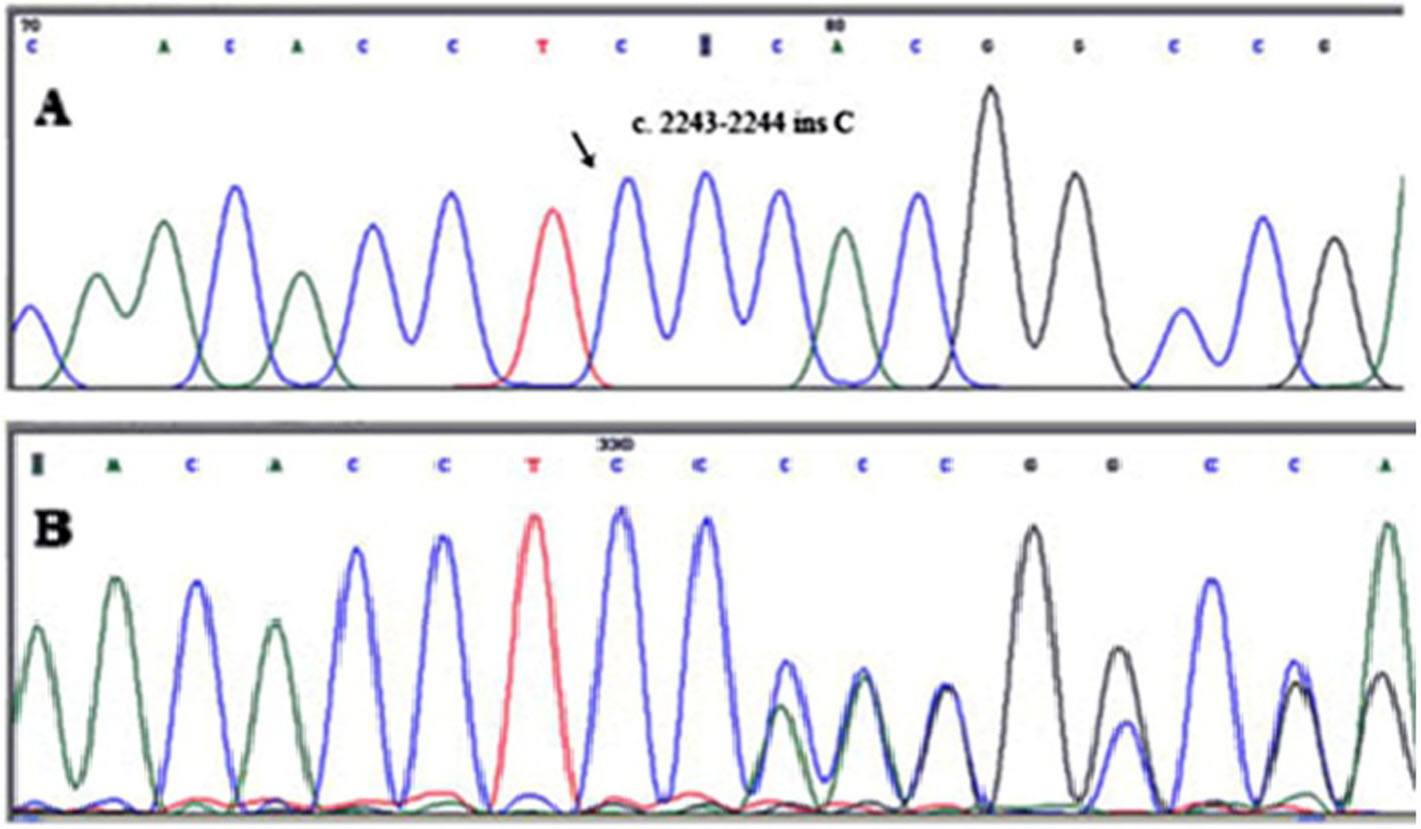
*WFS1* gene novel mutation in family II. **a** c.2243-2244insC electropherogram in the patient (III:7) and **b** His heterozygote parent (II:7)

In the patients from the second family, DNA sequencing revealed homozygosity for a frameshift mutation, c.2243-2244insC (T749Hfs*10). This mutation causes termination signal at codon 758 and produces a truncated protein that seems to be demolished after translation. Details of pathogenic analysis are provided in Additional file 1. This mutation is located in exon 8 of *WFS1*. In both families, patients were homozygous for this mutation but their parents and healthy siblings were either heterozygous or homozygous for the wild type allele (Fig. 5).

Both of the mutations were absent from SNP database version 147, 1000 genomes project, exome aggregation consortium and NHLBI exome sequencing project. Also mutations were predicted to be disease causing by MutationTaster. According to ACMG guideline, both variants are considered to be pathogenic.

Table 2 shows summary of clinical and molecular findings in the mentioned families.

**Table 2:** Summary of clinical and molecular findings in the mentioned families

Family Number		Mutation Position	Nucleotide change	Amino acid Change	Clinical findings
I	III:8	Exon 8	c.1232_1233delCT	S411 fs*541	Diabetes mellitus, bilateral optic atrophy, decreased vision, diabetes insipidus, hydronephrosis, atonic bladder, bilateral high-frequency sensorineural hearing loss
	III:9				Diabetes mellitus, decreased vision, bilateral optic atrophy, hydronephrosis, severe neurological problems
	IV:1				Hydroureteronephrosis, diabetes mellitus, vesicoureteral reflux, neuropathic bladder
II	III:7	Exon 8	c.2243-2244insC	T749Hfs*10	Diabetes mellitus, progressive visual loss, optic atrophy, bilateral hydronephrosis, neurogenic bladder
	III:3 and III:5				Diabetes mellitus, gradual loss of visual acuity, severe chronic kidney disease

## Discussion and conclusion

WS is a rare autosomal recessive neurodegenerative disorder that might represent a considerable fraction of diabetes especially among countries in the consanguinity belt. In this genetic study, we report clinical and molecular findings of nine patients from two large families.

In this study, a novel frameshift homozygous pathogenic variant leading to a familial case of WS was introduced. An 18-year-old male was referred to our clinic which had been diagnosed with T2DM and polyuria at the age of 5 years. His sister was also the known case of WS with the first diagnosis of T2DM at the age of seven-year-old. In a Sicilian district study, T2DM was diagnosed in 11 out of 12 WS patients under-10-year-old [20]. Interestingly, in both studies patients had shown T2DM characteristics except for the earlier commencement of manifestation and had been misdiagnosed of T2DM before their later WS diagnosis confirmation.

In the present study, we describe clinical manifestations and genetic analysis in a large family with 5 WS patients and with the high prevalence of diabetes among other members with heterozygous genotype. Increasing evidence indicates that ER stress and ER dysfunction play important roles in the pathogenesis of common diseases, such as T1DM and T2DM, as well as multiple neurodegenerative diseases [21]. Comprehensive studies have been done to find association between this gene and T2DM [22–24]. Genome-wide association (GWA) studies have reported *WFS1* among other genes to be associated with T2DM [24]. Obligate carriers have an increased prevalence of T2DM and deafness in some but not all studies. We found some *WFS1* genotype association with T2DM that was previously reported in several UK population and Ashkenazi Jews [23, 24 [24]] (Table 1). Confirmation in a larger sample set and meta-analyses across studies will be important to help determine the role of these variants.

In our study, we found one pathogenic mutation with important consequence for WFS1 structure and function resulting in a premature stop codon at residue 541, removing at least 349 amino (S411fsX541). The inheritance pattern of this mutation was compatible with autosomal recessive manner.

Out of 225 mutations reported for the gene, seventy-seven are small deletion based on WFS1 Gene Mutation and polymorphism Database (URL:http://www.khri.med.umich.edu/research/lesperance_lab/low_freq.php). Interestingly, in the same region that the novel mutation is found, a 4-bp deletion (c.1230_1233delCTCT) has been reported by Colosimo et al. in one patient. The variant creates a frameshift in the coding region of *WFS1*, causing a premature stop codon at residue 441 in compound heterozygosity with an in-frame deletion (c.1546delTTC), and lead to the deletion of a phenylalanine residue (F516del). The parents were non-consanguineous. The patient had developed T2DM at the age of three and optic atrophy at 10 years of age. She also had primary hypogonadism [20]. D’Annunzio et al. reported an Italian female with the same mutation (c.1230_1233delCTCT) which was homozygously inherited and showed all the clinical features of the syndrome [12]. Tessa et al. performed a study for identification of novel *WFS1* mutations in Italian children with WS and found the same mutation [21]. In the French population, two other mutations c.1232_1233delCT and c.1230_1233delCTCT were detected in the studied patients. In these patients, the mean ages of onset for T2DM and optic atrophy were 6 and 11 years old, respectively [22]. Van ven Ouwel et al. reported a 35-year-old woman who developed T2DM at the age of four and optic atrophy at the age of eight. She also presented other manifestations such as peripheral neuropathy and irritable bowel syndrome. They detected a heterozygous 4-bp deletion in a Dutch family (patient and her mother) leading to a frameshift at amino acid residue 410 which resulted in a pre-mature stop protein. She also inherited an allele with 15-bp deletion (1515- 1530del15nt) in exon 8 from her father [15]. In view of the findings mentioned above and the presence of a run of repeated CT bases (TTCCTGCTCTCTGTCTTC) (Fig. 4), the region of exon eight of *WFS1* gene can be considered to be a mutation hotspot posing the risk of strand slippage. Simple repetitive DNA sequences are a widespread and abundant feature of genomic DNA. Illegitimate base pairing in regions of repetitive DNA during replication, coupled with inadequate DNA mismatch repair systems, can produce deletions or insertions of repeat units [25].

All the five studied patients result from consanguineous marriages and with a family history of T2DM. Therefore, it would be wise to consider the syndrome as a possible etiology when dealing with childhood T2DM cases associated with other WS-related symptoms. In brief, one novel pathogenic variant was identified in this study. The variant is in exon 8 of the *WFS1* gene which causes the insertion of serine in the polypeptide chain and termination at codon 758 leading to a truncated protein that might be degraded post-translationally.

Clinical demonstrations were described in the patients. These findings have implications for genetic counseling for future families affected by these mutations and enable us to perform carrier detection, prenatal diagnosis and genotype-phenotype correlation for the family members. Molecular study of more Iranian WS patients is needed to determine the frequency and origin of the mutation.

## Supplementary information

**Additional file 1.** Details of pathogenic analysis.

## Data Availability

Data sharing is not applicable to this article as no datasets were generated or analysed during the current study.

## Abbreviations

GWA: Genome-wide association
T2DM: type-2 diabetes mellitus
VCUG: voiding cystourethrogram
WS: Wolfram syndrome
