# Correction to: Clinical and genetic analysis of two wolfram syndrome families with high occurrence of wolfram syndrome and diabetes type II: a case report

**DOI:** 10.1186/s12881-020-0980-y

**Published:** 2020-03-20

**Authors:** Maryam Sobhani, Mohammad Amin Tabatabaiefar, Soudeh Ghafouri-Fard, Asadollah Rajab, Asal Hojjat, Abdol-Mohammad Kajbafzadeh, Mohammad Reza Noori-Daloii

**Affiliations:** 1 Blood Transfusion Research Center, High Institute for Research and Education in Transfusion Medicine, Tehran, Iran; 2 Department of Genetics and Molecular Biology, School of Medicine, Isfahan University of Medical Sciences, Isfahan, Iran; 3 Pediatric Inherited Diseases Research Center, Research Institute for Primordial Prevention of Non-Communicable Disease, Isfahan University of Medical Sciences, Isfahan, Iran; 4 Department of Medical Genetics, Shahid Beheshti University of Medical Sciences, Tehran, Iran; 5 Iranian Diabetes Society, Tehran, Iran; 6 Pediatric Urology Research Center, Department of Pediatric Urology, Children’s Hospital Medical Center, Tehran University of Medical Sciences, Tehran, Iran; 7 Department of Medical Genetics, School of Medicine, Tehran University of Medical Sciences, Tehran, Iran

**Correction to: BMC Med Genet**

**https://doi.org/10.1186/s12881-020-0950-4**

Following publication of the original article [1], the authors flagged that the name of ‘Asal Hojjat’ was misspelled; the name had been spelled as ‘Asal Hojat’.

The spelling has since been corrected in the original article and is included in this correction.

The authors apologize for any inconvenience caused.
