# Supplementary material for: Clinical and genetic analysis of two wolfram syndrome families with high occurrence of wolfram syndrome and diabetes type II: a case report
Source: BMC Med Genet. 2020 Jan 14;21:13. doi: 10.1186/s12881-020-0950-4 (PMC6961406; doi:10.1186/s12881-020-0950-4)
Supplement: Supplementary file 1 — Additional file 1. Details of pathogenic analysis. [file 12881_2020_950_MOESM1_ESM.docx]

|  | First family |  |
| --- | --- | --- |

| **Prediction** | **disease causing** | **Model: *complex_aae*, prob: 1**     ([explain](http://doro.charite.de/MutationTaster/info/documentation.html#bayes)) |
| --- | --- | --- |
| **Summary** | - **amino acid sequence changed** - **frameshift** - **known disease mutation at this position (HGMD CD050484)** - **protein features (might be) affected** - **splice site changes** - **truncated protein (might cause NMD)** | [hyperlink](http://www.mutationtaster.org/cgi-bin/MutationTaster/MutationTaster69.cgi?sequence_snippet=CTGCTCT%5bCT/-%5dGTC&transcript_stable_id_text=ENST00000226760&gene=wfs1&sequence_type=cDNA&nt_alignment=1) |
| **analysed issue** | **analysis result** | |
| name of alteration | no title | |
| alteration (phys. location) | chr4:6302754_6302755delCT | |
| HGNC symbol | [WFS1](http://www.genedistiller.org/cgi-bin/GeneDistiller_results.cgi?show_interaction=1&show_hpo=1&show_pathways=1&txid=9606&order=start_pos&show_generifs=1&show_mgd_phenotypes=1&show_GO=1&show_pfam=1&show_transcripts=1&show_omim=1&show_synonyms=1&show_interpro=1&show_paralogs=1&show_proteinfamilies=1&genesymbol=WFS1) | |
| Ensembl transcript ID | [ENST00000226760](http://grch37.ensembl.org/Homo_sapiens/transview?db=core;transcript=ENST00000226760) | |
| Genbank transcript ID | [NM_001145853](http://www.ncbi.nlm.nih.gov/nuccore/NM_001145853) | |
| UniProt peptide | [O76024](http://www.uniprot.org/uniprot/O76024) | |
| alteration type | deletion | |
| alteration region | CDS | |
| DNA changes | c.1232_1233delCT cDNA.1402_1403delCT g.31179_31180delCT | |
| AA changes | S411Cfs*131 | |
| position(s) of altered AA if AA alteration in CDS | 411 (frameshift or PTC - further changes downstream) | |
| frameshift | yes | |
| known variant | Variant was neither found in ExAC nor 1000G. [Search ExAC.](http://exac.broadinstitute.org/variant/4-6302753-TCT-T)  known disease mutation at this position, [please check HGMD for details](http://www.hgmd.cf.ac.uk/ac/gene.php?gene=WFS1&accession=CD050484) (HGMD ID CD050484) | |
| regulatory features | H3K36me3, Histone, Histone 3 Lysine 36 Tri-Methylation H3K27me3, Histone, Histone 3 Lysine 27 Tri-Methylation | |
| phyloP / phastCons | \|  \| PhyloP \| PhastCons \| \| --- \| --- \| --- \| \| (flanking) \| 2.884 \| 0.993 \| \|  \| 5.483 \| 0.994 \| \|  \| -4.532 \| 0.001 \| \| (flanking) \| 5.483 \| 0.998 \|   [explain score(s)](http://www.mutationtaster.org/info/documentation.html#phylop) and/or inspect your position(s) in [in UCSC Genome Browser](http://genome.ucsc.edu/cgi-bin/hgTracks?org=Human&db=hg19&position=chr4:6302753-6302756) | |
| splice sites | \| effect \| gDNA position \| score \| wt detection sequence \| exon-intron border \| \| --- \| --- \| --- \| --- \| --- \| \| Acc increased \| 31188 \| wt: 0.71 / mu: 0.80 \| wt: TTTCCTGCTCTCTGTCTTCTTCGTCATCTTCTCCTTCCCCA mu: CATTTCCTGCTCTGTCTTCTTCGTCATCTTCTCCTTCCCCA \| tctt\|CGTC \| \| Acc marginally increased \| 31187 \| wt: 0.8093 / mu: 0.8686 (marginal change - not scored) \| wt: ATTTCCTGCTCTCTGTCTTCTTCGTCATCTTCTCCTTCCCC mu: CCATTTCCTGCTCTGTCTTCTTCGTCATCTTCTCCTTCCCC \| ttct\|TCGT \| \| Acc marginally increased \| 31189 \| wt: 0.9780 / mu: 0.9890 (marginal change - not scored) \| wt: TTCCTGCTCTCTGTCTTCTTCGTCATCTTCTCCTTCCCCAT mu: ATTTCCTGCTCTGTCTTCTTCGTCATCTTCTCCTTCCCCAT \| cttc\|GTCA \| \| Acc increased \| 31179 \| wt: 0.31 / mu: 0.55 \| wt: CTATGCCCATTTCCTGCTCTCTGTCTTCTTCGTCATCTTCT mu: CCCTATGCCCATTTCCTGCTCTGTCTTCTTCGTCATCTTCT \| tctc\|TGTC \| \| Acc marginally increased \| 31180 \| wt: 0.8078 / mu: 0.8880 (marginal change - not scored) \| wt: TATGCCCATTTCCTGCTCTCTGTCTTCTTCGTCATCTTCTC mu: CCTATGCCCATTTCCTGCTCTGTCTTCTTCGTCATCTTCTC \| ctct\|GTCT \| \| Acc gained \| 31191 \| 0.45 \| mu: TTCCTGCTCTGTCTTCTTCGTCATCTTCTCCTTCCCCATCG \| gtca\|TCTT \| | |
| distance from splice site | 371 | |
| Kozak consensus sequence altered? | N/A | |
| conservation protein level for non-synonymous changes | \| species \| match \| gene \| aa \| alignment \| \| \| \| \| \| \| \| \| \| \| \| \| \| \| \| \| \| \| \| \| \| \| \| \| \| \| \| \| \| \| \| \| \| \| \| \| \| \| \| \| \| \| \| \| \| \| \| \| \| \| \| \| \| \| \| \| \| \| --- \| --- \| --- \| --- \| --- \| --- \| --- \| --- \| --- \| --- \| --- \| --- \| --- \| --- \| --- \| --- \| --- \| --- \| --- \| --- \| --- \| --- \| --- \| --- \| --- \| --- \| --- \| --- \| --- \| --- \| --- \| --- \| --- \| --- \| --- \| --- \| --- \| --- \| --- \| --- \| --- \| --- \| --- \| --- \| --- \| --- \| --- \| --- \| --- \| --- \| --- \| --- \| --- \| --- \| --- \| --- \| --- \| --- \| --- \| --- \| --- \| --- \| \| Human \|  \|  \| 411 \| N \| H \| L \| E \| P \| Y \| A \| H \| F \| L \| L \| S \| V \| F \| F \| V \| I \| F \| S \| F \| P \| I \| A \| S \| K \| D \| C \| I \| P \| C \| S \| E \| L \| A \| V \| I \| T \| G \| F \| F \| T \| V \| T \| S \| Y \| L \| S \| L \| S \| T \| H \| A \| E \| P \| Y \| T \| R \| R \| ALATEVTAGLLSLLPSMPLNWPYLKVLGQTFITVPVGHLVVLNVSVPCLLYVYLLYLF \| \| mutated \| no alignment \|  \| n/a \|  \|  \|  \|  \|  \|  \|  \|  \|  \|  \|  \|  \|  \|  \|  \|  \|  \|  \|  \|  \|  \|  \|  \|  \|  \|  \|  \|  \|  \|  \|  \|  \|  \|  \|  \|  \|  \|  \|  \|  \|  \|  \|  \|  \|  \|  \|  \|  \|  \|  \|  \|  \|  \|  \|  \|  \|  \|  \|  \| \| Ptroglodytes \| no homologue \|  \|  \|  \|  \|  \|  \|  \|  \|  \|  \|  \|  \|  \|  \|  \|  \|  \|  \|  \|  \|  \|  \|  \|  \|  \|  \|  \|  \|  \|  \|  \|  \|  \|  \|  \|  \|  \|  \|  \|  \|  \|  \|  \|  \|  \|  \|  \|  \|  \|  \|  \|  \|  \|  \|  \|  \|  \|  \|  \|  \|  \| \| Mmulatta \| partly conserved \| [ENSMMUG00000013414](http://grch37.ensembl.org/Macaca_mulatta/Gene/Summary?db=core;g=ENSMMUG00000013414) \| 411 \| N \| H \| L \| E \| P \| Y \| A \| H \| F \| L \| L \| **S** \| **V** \| **V** \| **F** \| **V** \| **I** \| **F** \| **S** \| **F** \| **P** \| **I** \| **A** \| **S** \| **K** \| **D** \| **C** \| **I** \| **P** \| **C** \| **S** \| **E** \| **L** \| **A** \| **V** \| **I** \| **A** \| **G** \| **F** \| **F** \| **T** \| **V** \| **T** \| **S** \| **Y** \| **L** \| **S** \| **L** \| **S** \| **T** \| **H** \| **A** \| **E** \| **P** \| **Y** \| **T** \| **R** \| **R** \| **ALATEVTAGLLSLLPSMPLNWPY** \| \| Fcatus \| partly conserved \| [ENSFCAG00000009675](http://grch37.ensembl.org/Felis_catus/Gene/Summary?db=core;g=ENSFCAG00000009675) \| 332 \| N \| H \| L \| E \| P \| Y \| V \| H \| F \| L \| L \| **S** \| **V** \| **F** \| **F** \| **V** \| **I** \| **F** \| **S** \| **F** \| **P** \| **V** \| **A** \| **S** \| **K** \| **D** \| **F** \| **I** \| **P** \| **C** \| **S** \| **E** \| **L** \| **A** \| **V** \| **V** \| **A** \| **I** \| **F** \| **F** \| **T** \| **A** \| **T** \| **S** \| **Y** \| **M** \| **S** \| **L** \| **S** \| **T** \| **S** \| **A** \| **E** \| **P** \| **Y** \| **T** \| **R** \| **R** \| **ALVTEVAAGLLSLLPSLPFDWHHLKLLGQTFVTVPLGHLVV** \| \| Mmusculus \| partly conserved \| [ENSMUSG00000039474](http://grch37.ensembl.org/Mus_musculus/Gene/Summary?db=core;g=ENSMUSG00000039474) \| 413 \| N \| H \| L \| E \| P \| Y \| I \| H \| F \| L \| L \| **S** \| **V** \| **V** \| **F** \| **V** \| **I** \| **F** \| **S** \| **F** \| **P** \| **L** \| **A** \| **S** \| **K** \| **D** \| **C** \| **I** \| **P** \| **C** \| **S** \| **E** \| **L** \| **A** \| **V** \| **I** \| **S** \| **T** \| **F** \| **F** \| **T** \| **V** \| **T** \| **S** \| **Y** \| **M** \| **S** \| **L** \| **S** \| **S** \| **S** \| **A** \| **E** \| **P** \| **Y** \| **T** \| **R** \| **R** \| **ALVTEVAAGLLSLLPTVPVD** \| \| Ggallus \| no homologue \|  \|  \|  \|  \|  \|  \|  \|  \|  \|  \|  \|  \|  \|  \|  \|  \|  \|  \|  \|  \|  \|  \|  \|  \|  \|  \|  \|  \|  \|  \|  \|  \|  \|  \|  \|  \|  \|  \|  \|  \|  \|  \|  \|  \|  \|  \|  \|  \|  \|  \|  \|  \|  \|  \|  \|  \|  \|  \|  \|  \|  \| \| Trubripes \| no homologue \|  \|  \|  \|  \|  \|  \|  \|  \|  \|  \|  \|  \|  \|  \|  \|  \|  \|  \|  \|  \|  \|  \|  \|  \|  \|  \|  \|  \|  \|  \|  \|  \|  \|  \|  \|  \|  \|  \|  \|  \|  \|  \|  \|  \|  \|  \|  \|  \|  \|  \|  \|  \|  \|  \|  \|  \|  \|  \|  \|  \|  \| \| Drerio \| partly conserved \| [ENSDARG00000062341](http://grch37.ensembl.org/Danio_rerio/Gene/Summary?db=core;g=ENSDARG00000062341) \| 563 \| N \| N \| L \| E \| Q \| Y \| L \| Y \| F \| M \| I \| **S** \| **V** \| **F** \| **F** \| **V** \| **I** \| **F** \| **T** \| **F** \| **P** \| **V** \| **A** \| **D** \| **K** \| **G** \| **W** \| **I** \| **P** \| **C** \| **S** \| **E** \| **L** \| **S** \| **T** \| **V** \| **A** \| **I** \| **F** \| **F** \| **T** \| **A** \| **L** \| **S** \| **Y** \| **M** \| **S** \| **L** \| **S** \| **P** \| **S** \| **A** \| **A** \| **A** \| **Y** \| **A** \| **R** \| **K** \| **ALFIEVASSLCALTSRLPKDMVTARMLGQIFT** \| \| Dmelanogaster \| partly conserved \| [FBgn0039003](http://grch37.ensembl.org/Drosophila_melanogaster/Gene/Summary?db=core;g=FBgn0039003) \| 370 \| N \| N \| M \| K \| P \| Y \| L \| Y \| F \| F \| C \| **A** \| **F** \| **I** \| **C** \| **N** \| **L** \| **I** \| **V** \| **Y** \| **P** \| **L** \| **V** \| **T** \| **D** \| **A** \| **W** \| **L** \| **P** \| **H** \| **S** \| **E** \| **L** \| **T** \| **I** \| **I** \| **S** \| **G** \| **A** \| **L** \| **T** \| **F** \| **I** \| **T** \| **M** \| **C** \| **V** \| **S** \| **M** \| **Y** \| **A** \| **S** \| **S** \| **H** \| **Q** \| **L** \| **P** \| **D** \| **WLVIVSFAVNVLAK** \| \| Celegans \| no homologue \|  \|  \|  \|  \|  \|  \|  \|  \|  \|  \|  \|  \|  \|  \|  \|  \|  \|  \|  \|  \|  \|  \|  \|  \|  \|  \|  \|  \|  \|  \|  \|  \|  \|  \|  \|  \|  \|  \|  \|  \|  \|  \|  \|  \|  \|  \|  \|  \|  \|  \|  \|  \|  \|  \|  \|  \|  \|  \|  \|  \|  \| \| Xtropicalis \| partly conserved \| [ENSXETG00000011603](http://grch37.ensembl.org/Xenopus_tropicalis/Gene/Summary?db=core;g=ENSXETG00000011603) \| 351 \| N \| H \| M \| E \| P \| Y \| L \| Y \| F \| L \| I \| **S** \| **V** \| **F** \| **F** \| **V** \| **I** \| **F** \| **S** \| **F** \| **P** \| **I** \| **T** \| **D** \| **R** \| **N** \| **W** \| **I** \| **P** \| **C** \| **S** \| **E** \| **L** \| **A** \| **T** \| **I** \| **S** \| **I** \| **F** \| **F** \| **T** \| **I** \| **T** \| **S** \| **Y** \| **L** \| **S** \| **L** \| **S** \| **T** \| **S** \| **A** \| **E** \| **P** \| **Y** \| **T** \| **R** \| **R** \| **ALMTEVAAGALSLLDHLPISLPYLNIFSKTFFTVPVGHFIIFHISV** \| | |
| protein features | \| start (aa) \| end (aa) \| feature \| details \|  \| \| --- \| --- \| --- \| --- \| --- \| \| 402 \| 422 \| TRANSMEM \| Helical; (Potential). \| lost \| \| 427 \| 447 \| TRANSMEM \| Helical; (Potential). \| lost \| \| 465 \| 485 \| TRANSMEM \| Helical; (Potential). \| lost \| \| 496 \| 516 \| TRANSMEM \| Helical; (Potential). \| lost \| \| 529 \| 549 \| TRANSMEM \| Helical; (Potential). \| lost \| \| 563 \| 583 \| TRANSMEM \| Helical; (Potential). \| lost \| \| 589 \| 609 \| TRANSMEM \| Helical; (Potential). \| lost \| \| 632 \| 652 \| TRANSMEM \| Helical; (Potential). \| lost \| \| 870 \| 890 \| TRANSMEM \| Helical; (Potential). \| lost \| \| 877 \| 886 \| COMPBIAS \| Poly-Phe. \| lost \| | |
| length of protein | strongly truncated protein, might cause NMD (-350 AA / more than 10% missing) | |
| AA sequence altered | yes | |
| position of stopcodon in wt / mu CDS | 2673 / 1623 | |
| position (AA) of stopcodon in wt / mu AA sequence | 891 / 541 | |
| position of stopcodon in wt / mu cDNA | 2843 / 1793 | |
| poly(A) signal | N/A | |
| conservation nucleotide level for all changes - no scoring up to now | \| species \| match \| gene \| aa \| alignment \| \| \| \| \| \| \| \| \| \| \| \| \| \| \| \| \| \| \| \| \| \| \| \| \| \| --- \| --- \| --- \| --- \| --- \| --- \| --- \| --- \| --- \| --- \| --- \| --- \| --- \| --- \| --- \| --- \| --- \| --- \| --- \| --- \| --- \| --- \| --- \| --- \| --- \| --- \| --- \| --- \| --- \| \| Human \|  \|  \| 31179 \| T \| T \| T \| C \| C \| T \| G \| C \| T \| C \| T \| C \| T \| G \| T \| C \| T \| T \| C \| T \| T \| C \| G \| T \| C \| \| mutated \| not conserved \|  \| 31179 \| t \| t \| t \| c \| c \| t \| g \| c \| t \| c \| t \| **-** \| **-** \| g \| t \| c \| t \| t \| c \| t \| t \| c \| g \| t \|  \| \| Ptroglodytes \| no homologue \|  \|  \|  \|  \|  \|  \|  \|  \|  \|  \|  \|  \|  \|  \|  \|  \|  \|  \|  \|  \|  \|  \|  \|  \|  \|  \|  \| \| Mmulatta \| all identical \| [ENSMMUG00000013414](http://grch37.ensembl.org/Macaca_mulatta/Gene/Summary?db=core;g=ENSMMUG00000013414) \| 34765 \| c \| t \| t \| c \| c \| t \| g \| c \| t \| c \| t \| **c** \| **t** \| g \| t \| t \| g \| t \| c \| t \| t \| c \| g \| t \|  \| \| Fcatus \| partly conserved \| [ENSFCAG00000009675](http://grch37.ensembl.org/Felis_catus/Gene/Summary?db=core;g=ENSFCAG00000009675) \| 15293 \| c \| t \| t \| c \| c \| t \| g \| c \| t \| g \| t \| **c** \| **c** \| g \| t \| c \| t \| t \| c \| t \| t \| c \| g \| t \|  \| \| Mmusculus \| partly conserved \| [ENSMUSG00000039474](http://grch37.ensembl.org/Mus_musculus/Gene/Summary?db=core;g=ENSMUSG00000039474) \| 22675 \| c \| t \| t \| c \| c \| t \| a \| c \| t \| g \| t \| **c** \| **a** \| g \| t \| c \| g \| t \| c \| t \| t \| t \| g \| t \|  \| \| Ggallus \| no alignment \| [ENSGALG00000015529](http://grch37.ensembl.org/Gallus_gallus/Gene/Summary?db=core;g=ENSGALG00000015529) \| n/a \|  \|  \|  \|  \|  \|  \|  \|  \|  \|  \|  \|  \|  \|  \|  \|  \|  \|  \|  \|  \|  \|  \|  \|  \|  \| \| Trubripes \| no alignment \| [ENSTRUG00000010741](http://grch37.ensembl.org/Takifugu_rubripes/Gene/Summary?db=core;g=ENSTRUG00000010741) \| n/a \|  \|  \|  \|  \|  \|  \|  \|  \|  \|  \|  \|  \|  \|  \|  \|  \|  \|  \|  \|  \|  \|  \|  \|  \|  \| \| Drerio \| no alignment \| [ENSDARG00000062341](http://grch37.ensembl.org/Danio_rerio/Gene/Summary?db=core;g=ENSDARG00000062341) \| n/a \|  \|  \|  \|  \|  \|  \|  \|  \|  \|  \|  \|  \|  \|  \|  \|  \|  \|  \|  \|  \|  \|  \|  \|  \|  \| \| Dmelanogaster \| no alignment \| [FBgn0039003](http://grch37.ensembl.org/Drosophila_melanogaster/Gene/Summary?db=core;g=FBgn0039003) \| n/a \|  \|  \|  \|  \|  \|  \|  \|  \|  \|  \|  \|  \|  \|  \|  \|  \|  \|  \|  \|  \|  \|  \|  \|  \|  \| \| Celegans \| no homologue \|  \|  \|  \|  \|  \|  \|  \|  \|  \|  \|  \|  \|  \|  \|  \|  \|  \|  \|  \|  \|  \|  \|  \|  \|  \|  \|  \| \| Xtropicalis \| no alignment \| [ENSXETG00000011603](http://grch37.ensembl.org/Xenopus_tropicalis/Gene/Summary?db=core;g=ENSXETG00000011603) \| n/a \|  \|  \|  \|  \|  \|  \|  \|  \|  \|  \|  \|  \|  \|  \|  \|  \|  \|  \|  \|  \|  \|  \|  \|  \|  \| | |
| position of start ATG in wt / mu cDNA | 171 / 171 | |
| chromosome | 4 | |
| strand | 1 | |
| last intron/exon boundary | 1032 | |
| theoretical NMD boundary in CDS | 811 | |
| length of CDS | 2673 | |
| coding sequence (CDS) position | 1231 / 1234 | |
| cDNA position (for ins/del: last normal base / first normal base) | 1401 / 1404 | |
| gDNA position (for ins/del: last normal base / first normal base) | 31178 / 31181 | |
| chromosomal position (for ins/del: last normal base / first normal base) | 6302753 / 6302756 | |
| original gDNA sequence snippet | CTATGCCCATTTCCTGCTCT**CT**GTCTTCTTCGTCATCTTCTC | |
| altered gDNA sequence snippet | CTATGCCCATTTCCTGCTCTGTCTTCTTCGTCATCTTCTC | |
| original cDNA sequence snippet | CTATGCCCATTTCCTGCTCT**CT**GTCTTCTTCGTCATCTTCTC | |
| altered cDNA sequence snippet | CTATGCCCATTTCCTGCTCTGTCTTCTTCGTCATCTTCTC | |
| wildtype AA sequence | MDSNTAPLGP SCPQPPPAPQ PQARSRLNAT ASLEQERSER PRAPGPQAGP GPGVRDAAAP AEPQAQHTRS RERADGTGPT KGDMEIPFEE VLERAKAGDP KAQTEVGKHY LQLAGDTDEE LNSCTAVDWL VLAAKQGRRE AVKLLRRCLA DRRGITSENE REVRQLSSET DLERAVRKAA LVMYWKLNPK KKKQVAVAEL LENVGQVNEH DGGAQPGPVP KSLQKQRRML ERLVSSESKN YIALDDFVEI TKKYAKGVIP SSLFLQDDED DDELAGKSPE DLPLRLKVVK YPLHAIMEIK EYLIDMASRA GMHWLSTIIP THHINALIFF FIVSNLTIDF FAFFIPLVIF YLSFISMVIC TLKVFQDSKA WENFRTLTDL LLRFEPNLDV EQAEVNFGWN HLEPYAHFLL SVFFVIFSFP IASKDCIPCS ELAVITGFFT VTSYLSLSTH AEPYTRRALA TEVTAGLLSL LPSMPLNWPY LKVLGQTFIT VPVGHLVVLN VSVPCLLYVY LLYLFFRMAQ LRNFKGTYCY LVPYLVCFMW CELSVVILLE STGLGLLRAS IGYFLFLFAL PILVAGLALV GVLQFARWFT SLELTKIAVT VAVCSVPLLL RWWTKASFSV VGMVKSLTRS SMVKLILVWL TAIVLFCWFY VYRSEGMKVY NSTLTWQQYG ALCGPRAWKE TNMARTQILC SHLEGHRVTW TGRFKYVRVT DIDNSAESAI NMLPFFIGDW MRCLYGEAYP ACSPGNTSTA EEELCRLKLL AKHPCHIKKF DRYKFEITVG MPFSSGADGS RSREEDDVTK DIVLRASSEF KSVLLSLRQG SLIEFSTILE GRLGSKWPVF ELKAISCLNC MAQLSPTRRH VKIEHDWRST VHGAVKFAFD FFFFPFLSAA * | |
| mutated AA sequence | MDSNTAPLGP SCPQPPPAPQ PQARSRLNAT ASLEQERSER PRAPGPQAGP GPGVRDAAAP AEPQAQHTRS RERADGTGPT KGDMEIPFEE VLERAKAGDP KAQTEVGKHY LQLAGDTDEE LNSCTAVDWL VLAAKQGRRE AVKLLRRCLA DRRGITSENE REVRQLSSET DLERAVRKAA LVMYWKLNPK KKKQVAVAEL LENVGQVNEH DGGAQPGPVP KSLQKQRRML ERLVSSESKN YIALDDFVEI TKKYAKGVIP SSLFLQDDED DDELAGKSPE DLPLRLKVVK YPLHAIMEIK EYLIDMASRA GMHWLSTIIP THHINALIFF FIVSNLTIDF FAFFIPLVIF YLSFISMVIC TLKVFQDSKA WENFRTLTDL LLRFEPNLDV EQAEVNFGWN HLEPYAHFLL CLLRHLLLPH RQQGLHPLLG AGCHHRLLYR DQLPEPEHPC RALHAQGPGH RGHRRPAIAA ALHALELALP EGPWPDLHHR ACRPPGRPQR QRPVPALCLP ALSLLPHGTA EEFQGHLLLP CALPGVLHVV | |

Second family

| name of alteration | no title |
| --- | --- |
| alteration (phys. location) | chr4:6303765_6303766insC |
| HGNC symbol | [WFS1](http://www.genedistiller.org/cgi-bin/GeneDistiller_results.cgi?show_interaction=1&show_hpo=1&show_pathways=1&txid=9606&order=start_pos&show_generifs=1&show_mgd_phenotypes=1&show_GO=1&show_pfam=1&show_transcripts=1&show_omim=1&show_synonyms=1&show_interpro=1&show_paralogs=1&show_proteinfamilies=1&genesymbol=WFS1) |
| Ensembl transcript ID | [ENST00000226760](http://grch37.ensembl.org/Homo_sapiens/transview?db=core;transcript=ENST00000226760) |
| Genbank transcript ID | [NM_001145853](http://www.ncbi.nlm.nih.gov/nuccore/NM_001145853) |
| UniProt peptide | [O76024](http://www.uniprot.org/uniprot/O76024) |
| alteration type | insertion |
| alteration region | CDS |
| DNA changes | c.2243_2244insC cDNA.2413_2414insC g.32190_32191insC |
| AA changes | T749Hfs*10 |
| position(s) of altered AA if AA alteration in CDS | 749 (frameshift or PTC - further changes downstream) |
| frameshift | yes |
| known variant | Variant was neither found in ExAC nor 1000G. [Search ExAC.](http://exac.broadinstitute.org/variant/4-6303765-C-CC) |
| regulatory features | H3K36me3, Histone, Histone 3 Lysine 36 Tri-Methylation USF1, Transcription Factor, USF1 Transcription Factor Binding H3K27me3, Histone, Histone 3 Lysine 27 Tri-Methylation Gene Associated, Regulatory Feature, Gene associated regulatory feature |
| phyloP / phastCons | \|  \| PhyloP \| PhastCons \| \| --- \| --- \| --- \| \| (flanking) \| 2.24 \| 0.596 \| \| (flanking) \| 0.583 \| 0.605 \|   [explain score(s)](http://www.mutationtaster.org/info/documentation.html#phylop) and/or inspect your position(s) in [in UCSC Genome Browser](http://genome.ucsc.edu/cgi-bin/hgTracks?org=Human&db=hg19&position=chr4:6303765-6303766) |
| splice sites | \| effect \| gDNA position \| score \| wt detection sequence \| exon-intron border \| \| --- \| --- \| --- \| --- \| --- \| \| Acc gained \| 32193 \| 0.33 \| mu: AGCCCTGGCAACACCTCCCACGGCCGAGGAGGAGCTCTGTC \| ccca\|CGGC \| |
| distance from splice site | 1228 |
| Kozak consensus sequence altered? | N/A |
| conservation protein level for non-synonymous changes | \| species \| match \| gene \| aa \| alignment \| \| \| \| \| \| \| \| \| \| \| \| \| \| \| \| \| \| \| \| \| \| \| \| \| \| \| \| \| \| \| \| \| \| \| \| \| \| \| \| \| \| \| \| \| \| \| \| \| \| \| \| \| \| \| \| \| \| \| --- \| --- \| --- \| --- \| --- \| --- \| --- \| --- \| --- \| --- \| --- \| --- \| --- \| --- \| --- \| --- \| --- \| --- \| --- \| --- \| --- \| --- \| --- \| --- \| --- \| --- \| --- \| --- \| --- \| --- \| --- \| --- \| --- \| --- \| --- \| --- \| --- \| --- \| --- \| --- \| --- \| --- \| --- \| --- \| --- \| --- \| --- \| --- \| --- \| --- \| --- \| --- \| --- \| --- \| --- \| --- \| --- \| --- \| --- \| --- \| --- \| --- \| \| Human \|  \|  \| 749 \| A \| Y \| P \| A \| C \| S \| P \| G \| N \| T \| S \| T \| A \| E \| E \| E \| L \| C \| R \| L \| K \| L \| L \| A \| K \| H \| P \| C \| H \| I \| K \| K \| F \| D \| R \| Y \| K \| F \| E \| I \| T \| V \| G \| M \| P \| F \| S \| S \| G \| A \| D \| G \| S \| R \| S \| R \| E \| E \| DDVTKDIVLRASSEFKSVLLSLRQGSLIEFSTILEGRLGSKWPVFELKAISCLNCMAQLSPTRRHVKIEHDWRSTVHGAV \| \| mutated \| no alignment \|  \| n/a \|  \|  \|  \|  \|  \|  \|  \|  \|  \|  \|  \|  \|  \|  \|  \|  \|  \|  \|  \|  \|  \|  \|  \|  \|  \|  \|  \|  \|  \|  \|  \|  \|  \|  \|  \|  \|  \|  \|  \|  \|  \|  \|  \|  \|  \|  \|  \|  \|  \|  \|  \|  \|  \|  \|  \|  \|  \|  \|  \| \| Ptroglodytes \| no homologue \|  \|  \|  \|  \|  \|  \|  \|  \|  \|  \|  \|  \|  \|  \|  \|  \|  \|  \|  \|  \|  \|  \|  \|  \|  \|  \|  \|  \|  \|  \|  \|  \|  \|  \|  \|  \|  \|  \|  \|  \|  \|  \|  \|  \|  \|  \|  \|  \|  \|  \|  \|  \|  \|  \|  \|  \|  \|  \|  \|  \|  \| \| Mmulatta \| partly conserved \| [ENSMMUG00000013414](http://grch37.ensembl.org/Macaca_mulatta/Gene/Summary?db=core;g=ENSMMUG00000013414) \| 749 \| A \| Y \| P \| A \| C \| S \| P \| G \| N \| T \| S \| **T** \| **A** \| **E** \| **E** \| **E** \| **L** \| **C** \| **R** \| **L** \| **K** \| **L** \| **L** \| **A** \| **K** \| **H** \| **P** \| **C** \| **H** \| **I** \| **K** \| **K** \| **F** \| **D** \| **R** \| **Y** \| **K** \| **F** \| **E** \| **I** \| **T** \| **V** \| **G** \| **M** \| **P** \| **F** \| **S** \| **S** \| **G** \| **A** \| **N** \| **S** \| **S** \| **R** \| **G** \| **R** \| **E** \| **E** \| **DDVTKDIVLRASSEFKSVLLSLRQGSVIEFSTILEGRLGSKWPVF** \| \| Fcatus \| partly conserved \| [ENSFCAG00000009675](http://grch37.ensembl.org/Felis_catus/Gene/Summary?db=core;g=ENSFCAG00000009675) \| 670 \|  \| Y \| P \| A \| C \| S \| P \| G \| T \| A \| P \| **T** \| **A** \| **E** \| **E** \| **E** \| **L** \| **C** \| **R** \| **L** \| **K** \| **L** \| **L** \| **A** \| **K** \| **H** \| **P** \| **C** \| **H** \| **I** \| **K** \| **K** \| **F** \| **D** \| **R** \| **Y** \| **K** \| **F** \| **E** \| **I** \| **T** \| **V** \| **G** \| **M** \| **P** \| **Y** \| **-** \| **S** \| **G** \| **A** \| **N** \| **G** \| **S** \| **R** \| **S** \| **P** \| **E** \| **E** \| **DDVTKDLVLRASGEFKDVLLSLRQGSLIEFSTVLEGRLGSKWPVFELKAIRCLNCMAQLTPAR** \| \| Mmusculus \| partly conserved \| [ENSMUSG00000039474](http://grch37.ensembl.org/Mus_musculus/Gene/Summary?db=core;g=ENSMUSG00000039474) \| 751 \| A \| Y \| P \| S \| C \| S \| S \| G \| N \| T \| S \| **T** \| **A** \| **E** \| **E** \| **E** \| **L** \| **C** \| **R** \| **L** \| **K** \| **Q** \| **L** \| **A** \| **K** \| **H** \| **P** \| **C** \| **H** \| **I** \| **K** \| **K** \| **F** \| **D** \| **R** \| **Y** \| **K** \| **F** \| **E** \| **I** \| **T** \| **V** \| **G** \| **M** \| **P** \| **F** \| **-** \| **-** \| **G** \| **T** \| **N** \| **G** \| **N** \| **R** \| **G** \| **H** \| **E** \| **E** \| **DDITKDIVLRASSEFKDVLLNLRQGSLIEFSTILEGRLGSKW** \| \| Ggallus \| no homologue \|  \|  \|  \|  \|  \|  \|  \|  \|  \|  \|  \|  \|  \|  \|  \|  \|  \|  \|  \|  \|  \|  \|  \|  \|  \|  \|  \|  \|  \|  \|  \|  \|  \|  \|  \|  \|  \|  \|  \|  \|  \|  \|  \|  \|  \|  \|  \|  \|  \|  \|  \|  \|  \|  \|  \|  \|  \|  \|  \|  \|  \| \| Trubripes \| no homologue \|  \|  \|  \|  \|  \|  \|  \|  \|  \|  \|  \|  \|  \|  \|  \|  \|  \|  \|  \|  \|  \|  \|  \|  \|  \|  \|  \|  \|  \|  \|  \|  \|  \|  \|  \|  \|  \|  \|  \|  \|  \|  \|  \|  \|  \|  \|  \|  \|  \|  \|  \|  \|  \|  \|  \|  \|  \|  \|  \|  \|  \| \| Drerio \| partly conserved \| [ENSDARG00000062341](http://grch37.ensembl.org/Danio_rerio/Gene/Summary?db=core;g=ENSDARG00000062341) \| 901 \| V \| Y \| P \| K \| C \| E \| P \| Q \| N \| N \| SFPIVNVTQVTTGTVNTTILILR \| **Q** \| **E** \| **E** \| **E** \| **E** \| **L** \| **C** \| **R** \| **I** \| **K** \| **A** \| **Y** \| **A** \| **T** \| **H** \| **Q** \| **C** \| **H** \| **V** \| **K** \| **R** \| **F** \| **D** \| **S** \| **Y** \| **R** \| **F** \| **E** \| **V** \| **T** \| **V** \| **G** \| **M** \| **P** \| **-** \| **-** \| **-** \| **-** \| **V** \| **D** \| **G** \| **-** \| **-** \| **V** \| **T** \| **K** \| **V** \| **DNPAGDILLMASHEFRQVLLNLNPGSMVEFST** \| \| Dmelanogaster \| partly conserved \| [FBgn0039003](http://grch37.ensembl.org/Drosophila_melanogaster/Gene/Summary?db=core;g=FBgn0039003) \| 727 \|  \|  \|  \|  \|  \|  \|  \|  \|  \|  \| A \| **Q** \| **C** \| **E** \| **E** \| **W** \| **R** \| **S** \| **V** \| **F** \| **K** \| **T** \| **FNA** \| **Q** \| **S** \| **G** \| **S** \| **C** \| **T** \| **L** \| **Q** \| **R** \| **W** \| **N** \| **R** \| **Y** \| **E** \| **Y** \| **E** \| **L** \| **L** \| **V** \| **K** \| **V** \| **-** \| **-** \| **-** \| **-** \| **G** \| **T** \| **K** \| **R** \| **S** \| **G** \| **R** \| **L** \| **L** \| **G** \| **RSTTTDVILRAHHDFGNFTRLLSEGDVVLFYGILHNSRLLADNVQVKLKTIECVECRSR** \| \| Celegans \| no homologue \|  \|  \|  \|  \|  \|  \|  \|  \|  \|  \|  \|  \|  \|  \|  \|  \|  \|  \|  \|  \|  \|  \|  \|  \|  \|  \|  \|  \|  \|  \|  \|  \|  \|  \|  \|  \|  \|  \|  \|  \|  \|  \|  \|  \|  \|  \|  \|  \|  \|  \|  \|  \|  \|  \|  \|  \|  \|  \|  \|  \|  \| \| Xtropicalis \| partly conserved \| [ENSXETG00000011603](http://grch37.ensembl.org/Xenopus_tropicalis/Gene/Summary?db=core;g=ENSXETG00000011603) \| 689 \|  \|  \|  \|  \|  \|  \| P \| Q \| N \| V \| T \| **M** \| **E** \| **E** \| **E** \| **E** \| **L** \| **C** \| **R** \| **L** \| **K** \| **F** \| **L** \| **S** \| **K** \| **H** \| **S** \| **C** \| **H** \| **M** \| **-** \| **K** \| **F** \| **D** \| **R** \| **Y** \| **K** \| **F** \| **E** \| **I** \| **T** \| **V** \| **G** \| **M** \| **P** \| **F** \| **-** \| **N** \| **G** \| **V** \| **N** \| **A** \| **S** \| **K** \| **P** \| **I** \| **E** \| **E** \| **DDITKDIVLRASNEFKQVLLNLRQGSIIEFSTILEGRLGSKWPVFELKAISCLNCMAKLTPASRHVKI** \| |
| protein features | \| start (aa) \| end (aa) \| feature \| details \|  \| \| --- \| --- \| --- \| --- \| --- \| \| 870 \| 890 \| TRANSMEM \| Helical; (Potential). \| lost \| \| 877 \| 886 \| COMPBIAS \| Poly-Phe. \| lost \| |
| length of protein | strongly truncated protein, might cause NMD (-133 AA / more than 10% missing) |
| AA sequence altered | yes |
| position of stopcodon in wt / mu CDS | 2673 / 2274 |
| position (AA) of stopcodon in wt / mu AA sequence | 891 / 758 |
| position of stopcodon in wt / mu cDNA | 2843 / 2444 |
| poly(A) signal | N/A |
| conservation nucleotide level for all changes - no scoring up to now | \| species \| match \| gene \| aa \| alignment \| \| \| \| \| \| \| \| \| \| \| \| \| \| \| \| \| \| \| \| \| \| \| \| \| --- \| --- \| --- \| --- \| --- \| --- \| --- \| --- \| --- \| --- \| --- \| --- \| --- \| --- \| --- \| --- \| --- \| --- \| --- \| --- \| --- \| --- \| --- \| --- \| --- \| --- \| --- \| --- \| \| Human \|  \|  \| 32191 \| G \| G \| C \| A \| A \| C \| A \| C \| C \| T \| C \| C \| A \| C \| G \| G \| C \| C \| G \| A \| G \| G \| A \| G \| \| mutated \| n/a \|  \| 32191 \| g \| g \| c \| a \| a \| c \| a \| c \| c \| t \| c \| cc \| a \| c \| g \| g \| c \| c \| g \| a \| g \| g \|  \|  \| \| Ptroglodytes \| no homologue \|  \|  \|  \|  \|  \|  \|  \|  \|  \|  \|  \|  \|  \|  \|  \|  \|  \|  \|  \|  \|  \|  \|  \|  \|  \|  \| \| Mmulatta \| n/a \| [ENSMMUG00000013414](http://grch37.ensembl.org/Macaca_mulatta/Gene/Summary?db=core;g=ENSMMUG00000013414) \| 35777 \| g \| g \| c \| a \| a \| c \| a \| c \| c \| t \| c \| c \| a \| c \| g \| g \| c \| c \| g \| a \| g \| g \|  \|  \| \| Fcatus \| n/a \| [ENSFCAG00000009675](http://grch37.ensembl.org/Felis_catus/Gene/Summary?db=core;g=ENSFCAG00000009675) \| 16307 \| g \| g \| c \| a \| c \| g \| g \| c \| g \| c \| c \| c \| a \| c \| g \| g \| c \| c \| g \| a \| g \| g \|  \|  \| \| Mmusculus \| n/a \| [ENSMUSG00000039474](http://grch37.ensembl.org/Mus_musculus/Gene/Summary?db=core;g=ENSMUSG00000039474) \| 23687 \| g \| g \| t \| a \| a \| c \| a \| c \| g \| t \| c \| c \| a \| c \| g \| g \| c \| a \| g \| a \| g \| g \|  \|  \| \| Ggallus \| no alignment \| [ENSGALG00000015529](http://grch37.ensembl.org/Gallus_gallus/Gene/Summary?db=core;g=ENSGALG00000015529) \| n/a \|  \|  \|  \|  \|  \|  \|  \|  \|  \|  \|  \|  \|  \|  \|  \|  \|  \|  \|  \|  \|  \|  \|  \|  \| \| Trubripes \| no alignment \| [ENSTRUG00000010741](http://grch37.ensembl.org/Takifugu_rubripes/Gene/Summary?db=core;g=ENSTRUG00000010741) \| n/a \|  \|  \|  \|  \|  \|  \|  \|  \|  \|  \|  \|  \|  \|  \|  \|  \|  \|  \|  \|  \|  \|  \|  \|  \| \| Drerio \| no alignment \| [ENSDARG00000062341](http://grch37.ensembl.org/Danio_rerio/Gene/Summary?db=core;g=ENSDARG00000062341) \| n/a \|  \|  \|  \|  \|  \|  \|  \|  \|  \|  \|  \|  \|  \|  \|  \|  \|  \|  \|  \|  \|  \|  \|  \|  \| \| Dmelanogaster \| no alignment \| [FBgn0039003](http://grch37.ensembl.org/Drosophila_melanogaster/Gene/Summary?db=core;g=FBgn0039003) \| n/a \|  \|  \|  \|  \|  \|  \|  \|  \|  \|  \|  \|  \|  \|  \|  \|  \|  \|  \|  \|  \|  \|  \|  \|  \| \| Celegans \| no homologue \|  \|  \|  \|  \|  \|  \|  \|  \|  \|  \|  \|  \|  \|  \|  \|  \|  \|  \|  \|  \|  \|  \|  \|  \|  \|  \| \| Xtropicalis \| no alignment \| [ENSXETG00000011603](http://grch37.ensembl.org/Xenopus_tropicalis/Gene/Summary?db=core;g=ENSXETG00000011603) \| n/a \|  \|  \|  \|  \|  \|  \|  \|  \|  \|  \|  \|  \|  \|  \|  \|  \|  \|  \|  \|  \|  \|  \|  \|  \| |
| position of start ATG in wt / mu cDNA | 171 / 171 |
| chromosome | 4 |
| strand | 1 |
| last intron/exon boundary | 1032 |
| theoretical NMD boundary in CDS | 811 |
| length of CDS | 2673 |
| coding sequence (CDS) position | 2243 / 2244 |
| cDNA position (for ins/del: last normal base / first normal base) | 2413 / 2414 |
| gDNA position (for ins/del: last normal base / first normal base) | 32190 / 32191 |
| chromosomal position (for ins/del: last normal base / first normal base) | 6303765 / 6303766 |
| original gDNA sequence snippet | TGCAGCCCTGGCAACACCTCCACGGCCGAGGAGGAGCTCT |
| altered gDNA sequence snippet | TGCAGCCCTGGCAACACCTC**C**CACGGCCGAGGAGGAGCTCT |
| original cDNA sequence snippet | TGCAGCCCTGGCAACACCTCCACGGCCGAGGAGGAGCTCT |
| altered cDNA sequence snippet | TGCAGCCCTGGCAACACCTC**C**CACGGCCGAGGAGGAGCTCT |
| wildtype AA sequence | MDSNTAPLGP SCPQPPPAPQ PQARSRLNAT ASLEQERSER PRAPGPQAGP GPGVRDAAAP AEPQAQHTRS RERADGTGPT KGDMEIPFEE VLERAKAGDP KAQTEVGKHY LQLAGDTDEE LNSCTAVDWL VLAAKQGRRE AVKLLRRCLA DRRGITSENE REVRQLSSET DLERAVRKAA LVMYWKLNPK KKKQVAVAEL LENVGQVNEH DGGAQPGPVP KSLQKQRRML ERLVSSESKN YIALDDFVEI TKKYAKGVIP SSLFLQDDED DDELAGKSPE DLPLRLKVVK YPLHAIMEIK EYLIDMASRA GMHWLSTIIP THHINALIFF FIVSNLTIDF FAFFIPLVIF YLSFISMVIC TLKVFQDSKA WENFRTLTDL LLRFEPNLDV EQAEVNFGWN HLEPYAHFLL SVFFVIFSFP IASKDCIPCS ELAVITGFFT VTSYLSLSTH AEPYTRRALA TEVTAGLLSL LPSMPLNWPY LKVLGQTFIT VPVGHLVVLN VSVPCLLYVY LLYLFFRMAQ LRNFKGTYCY LVPYLVCFMW CELSVVILLE STGLGLLRAS IGYFLFLFAL PILVAGLALV GVLQFARWFT SLELTKIAVT VAVCSVPLLL RWWTKASFSV VGMVKSLTRS SMVKLILVWL TAIVLFCWFY VYRSEGMKVY NSTLTWQQYG ALCGPRAWKE TNMARTQILC SHLEGHRVTW TGRFKYVRVT DIDNSAESAI NMLPFFIGDW MRCLYGEAYP ACSPGNTSTA EEELCRLKLL AKHPCHIKKF DRYKFEITVG MPFSSGADGS RSREEDDVTK DIVLRASSEF KSVLLSLRQG SLIEFSTILE GRLGSKWPVF ELKAISCLNC MAQLSPTRRH VKIEHDWRST VHGAVKFAFD FFFFPFLSAA * |
| mutated AA sequence | MDSNTAPLGP SCPQPPPAPQ PQARSRLNAT ASLEQERSER PRAPGPQAGP GPGVRDAAAP AEPQAQHTRS RERADGTGPT KGDMEIPFEE VLERAKAGDP KAQTEVGKHY LQLAGDTDEE LNSCTAVDWL VLAAKQGRRE AVKLLRRCLA DRRGITSENE REVRQLSSET DLERAVRKAA LVMYWKLNPK KKKQVAVAEL LENVGQVNEH DGGAQPGPVP KSLQKQRRML ERLVSSESKN YIALDDFVEI TKKYAKGVIP SSLFLQDDED DDELAGKSPE DLPLRLKVVK YPLHAIMEIK EYLIDMASRA GMHWLSTIIP THHINALIFF FIVSNLTIDF FAFFIPLVIF YLSFISMVIC TLKVFQDSKA WENFRTLTDL LLRFEPNLDV EQAEVNFGWN HLEPYAHFLL SVFFVIFSFP IASKDCIPCS ELAVITGFFT VTSYLSLSTH AEPYTRRALA TEVTAGLLSL LPSMPLNWPY LKVLGQTFIT VPVGHLVVLN VSVPCLLYVY LLYLFFRMAQ LRNFKGTYCY LVPYLVCFMW CELSVVILLE STGLGLLRAS IGYFLFLFAL PILVAGLALV GVLQFARWFT SLELTKIAVT VAVCSVPLLL RWWTKASFSV VGMVKSLTRS SMVKLILVWL TAIVLFCWFY VYRSEGMKVY NSTLTWQQYG ALCGPRAWKE TNMARTQILC SHLEGHRVTW TGRFKYVRVT DIDNSAESAI NMLPFFIGDW MRCLYGEAYP ACSPGNTSHG RGGALSP* |
| speed | 0.23 s |
